# Combined pituitary hormone deficiency harboring *CHD7* gene missense mutation without CHARGE syndrome: a case report

**DOI:** 10.1186/s12902-023-01373-8

**Published:** 2023-05-25

**Authors:** Yoshinari Obata, Kana Takayama, Hideyuki Nishikubo, Aoki Tobimatsu, Izumi Matsuda, Yuhei Uehara, Yumiko Maruo, Hiroyuki Sho, Motohiro Kosugi, Tetsuyuki Yasuda

**Affiliations:** Department of Diabetes and Endocrinology, Osaka Police Hospital, 10-31 Kitayama-Cho, Tennojiku, Osaka 543-0035 Japan

**Keywords:** Combined pituitary hormone deficiency, CHD7, CHARGE syndrome, Case report

## Abstract

**Background:**

Heterozygous loss-of-function mutations in the chromodomain helicase DNA-binding protein 7 (*CHD7*) gene cause CHARGE syndrome characterized by various congenital anomalies. A majority of patients with CHARGE syndrome present with congenital hypogonadotropic hypogonadism (HH), and combined pituitary hormone deficiency (CPHD) can also be present. Whereas *CHD7* mutations have been identified in some patients with isolated HH without a diagnosis of CHARGE syndrome, it remains unclear whether *CHD7* mutations can be identified in patients with CPHD who do not fulfill the criteria for CHARGE syndrome.

**Case presentation:**

A 33-year-old woman was admitted to our hospital. She had primary amenorrhea and was at Tanner stage 2 for both pubic hair and breast development. She was diagnosed with CPHD (HH, growth hormone deficiency, and central hypothyroidism), and a heterozygous rare missense mutation (c.6745G > A, p.Asp2249Asn) in the *CHD7* gene was identified. Our conservation analysis and numerous in silico analyses suggested that this mutation had pathogenic potential. She had mild intellectual disability, a minor feature of CHARGE syndrome, but did not fulfill the criteria for CHARGE syndrome.

**Conclusions:**

We report a rare case of CPHD harboring *CHD7* mutation without CHARGE syndrome. This case provides valuable insights into phenotypes caused by *CHD7* mutations. *CHD7* mutations can have a continuous phenotypic spectrum depending on the severity of hypopituitarism and CHARGE features. Therefore, we would like to propose a novel concept of CHD7-associated syndrome.

## Background

Heterozygous loss-of-function mutations in the chromodomain helicase DNA-binding protein 7 (*CHD7*) gene constitute the major pathogenic cause of CHARGE syndrome [1, 2]. CHARGE syndrome is a rare disorder characterized by various congenital anomalies including coloboma of the eye, heart defects, choanal atresia, retardation of growth and development, genital hypoplasia, and ear abnormalities [3, 4]. Hypothalamic-pituitary dysfunction is common in CHARGE syndrome and approximately 60–80% of individuals with CHARGE syndrome present with congenital hypogonadotropic hypogonadism (HH) [5–11]. Combined pituitary hormone deficiency (CPHD) can also be present. Growth hormone (GH) and thyroid-stimulating hormone (TSH) deficiencies occur at rates of 9–34% [5–8] and 8–18% [6–9], respectively, and sporadic cases of adrenocorticotropic hormone (ACTH) deficiency have been reported [12, 13]. Rarely do structural pituitary abnormalities such as anterior pituitary hypoplasia occur in combination [13]. *CHD7* mutations have also been identified in 5–19% of patients with isolated HH who were not diagnosed with CHARGE syndrome [11, 14–20]. However, it remains unclear whether *CHD7* mutations can be detected in patients with CPHD who do not fulfill the criteria for CHARGE syndrome. Herein, we present a rare case of patient with CPHD, who despite not fulfilling the diagnostic criteria for CHARGE syndrome, presented with minor characteristics and a *CHD7* missense mutation with pathogenic potential.

## Case presentation

The patient provided written informed consent for the publication of this case report. A 33-year-old woman was admitted to our hospital for treatment of diabetes mellitus. She had been born full-term, with a weight of approximately 2800 g, with no perinatal or infantile developmental abnormalities although detailed information was unavailable. Around the age of 7 years, she had begun to experience a decline in academic performance and weight gain. She had been the first or second shortest in her class until approximately 15 years, after which her height begun to increase. At the age of 15 years, she visited a hospital with a chief complaint of amenorrhea. Further examination was not performed because her symptom had been attributed to obesity; however, she did not menstruate thereafter. At the age of 16 years, she got a job, but had to change employment frequently because she could not work for long periods at one job. At the age 33 years, she was incidentally diagnosed with diabetes at another clinic and referred to our hospital.

**Fig. 1:**
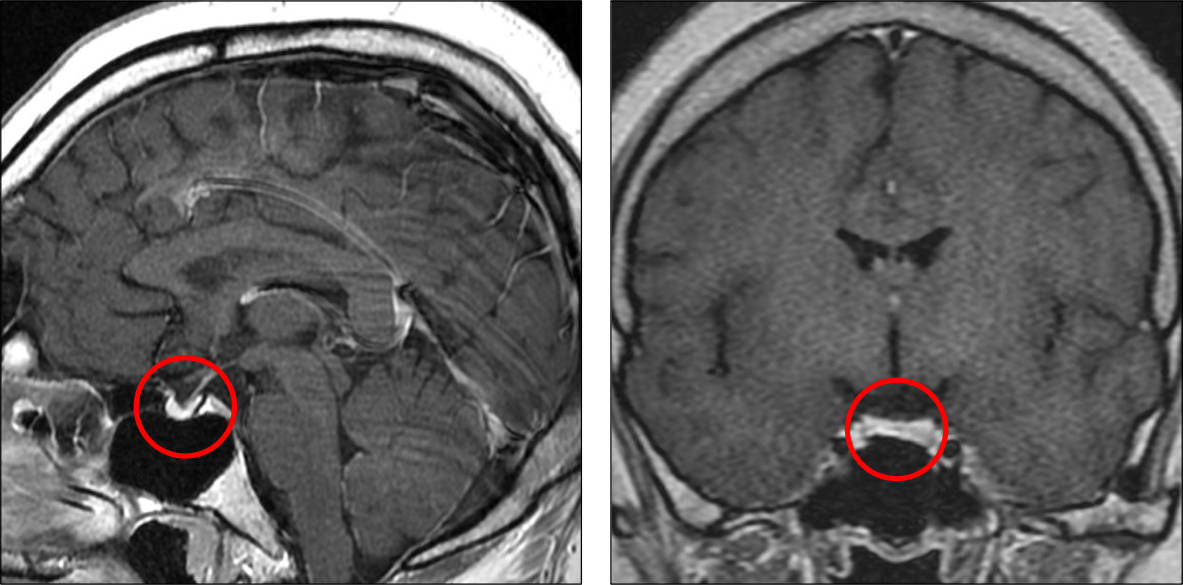
Gadolinium-enhanced brain magnetic resonance imaging showing anterior pituitary hypoplasia (circle)

**Fig. 2:**
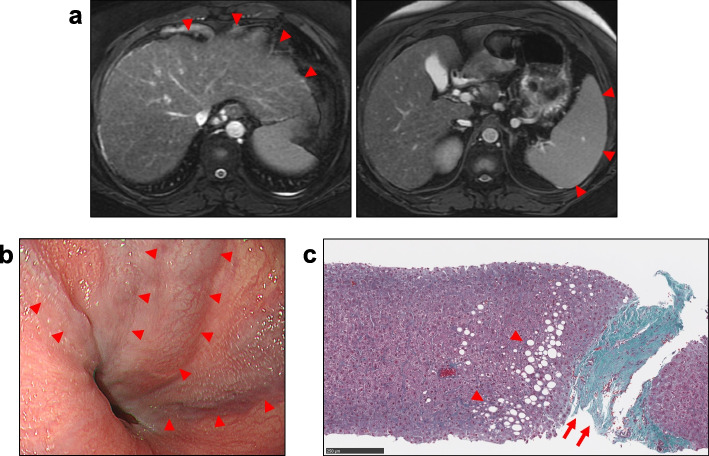
**a**: Abdominal magnetic resonance imaging showing morphological features characteristic of cirrhosis such as irregularity of the liver surface, enlargement of the left lobe, and splenomegaly (arrowhead). **b**: Esophagogastroduodenoscopy showing esophagus varices (arrowhead). **c**: Histological analysis of the liver with Masson’s trichrome staining showing severe fibrosis (arrow) and mild steatosis (arrowhead)

On admission, the patient’s height, weight, body mass index, waist circumference, and arm span were 161.1 cm, 97.1 kg, 37.4 kg/m^2^, 111.2 cm, and 160.0 cm, respectively. Her visceral fat area estimated via bioelectrical impedance analysis using EW-FA90 (Panasonic Corporation, Osaka, Japan) was 166 cm^2^. She had no history of head trauma or head surgery, and neither drank alcohol nor took any medications. Although her maternal grandmother had obesity and diabetes, she had no other family members with obesity, diabetes, or endocrinological disorders, and there were no consanguineous marriages in her family. She had primary amenorrhea and was at Tanner stage 2 for both pubic hair and breast development. Her intelligence quotient, as determined by the Wechsler Adult Intelligence Scale, fourth edition, was 60, indicating mild intellectual disability. Her laboratory findings are presented in Table 1. Her hemoglobin A1c level was 9.7%. She had thrombocytopenia, prolonged prothrombin time, and elevated liver fibrosis marker levels, suggestive of liver cirrhosis. Viral hepatitis, autoimmune hepatitis, primary biliary cholangitis, Wilson’s disease, and alcoholic hepatitis were unlikely differential diagnoses, suggesting that her liver dysfunction was caused by nonalcoholic steatohepatitis (NASH). Her endocrinological findings are presented in Tables 1 and 2. HH and growth hormone deficiency (GHD) were confirmed by comparing baseline hormonal levels and the results of stimulation tests. Central hypothyroidism (CH) was diagnosed due to low free thyroxine level and normal or mildly elevated TSH levels at the baseline, although the TSH level responded normally to thyrotropin-releasing hormone (TRH) stimulation. Plasma ACTH levels showed normal responses in both the insulin tolerance test (ITT) and corticotropin-releasing hormone (CRH) stimulation test. Cortisol response was almost normal in the rapid ACTH stimulation test but blunted in the ITT and CRH stimulation test. Based on these results and the absence of clinical signs of adrenal insufficiency, central adrenal insufficiency was not apparent. Her prolactin level responded normally to TRH stimulation. Thus, she was diagnosed with CPHD (combined HH, GHD, and CH). Brain magnetic resonance imaging (MRI) revealed anterior pituitary hypoplasia (Fig. 1). There were no other abnormal intracranial findings, including those of the posterior pituitary and pituitary stalk. Abdominal MRI showed morphological features of cirrhosis (Fig. 2a) and esophagogastroduodenoscopy (EGD) showed esophageal varices (Fig. 2b). Liver biopsy revealed severe fibrosis (Fig. 2c). Although there was no histological evidence of NASH, such as severe steatosis or ballooning, the clinical course and laboratory findings shown in Table 1 suggested that the liver cirrhosis was caused by burnt-out NASH. Based on these results, we diagnosed her with CPHD (combined HH, GHD, and CH) with comorbid diabetes, severe obesity, and liver cirrhosis probably due to NASH. She started treatment with recombinant GH, levothyroxine sodium, and estrogen/progesterone therapy. Her glycemic control was remarkably improved by treatment with linagliptin and empagliflozin.

**Table 1:** Laboratory characteristics of the patient at the time of admission

**Hematologic characteristics**				CPR	1.5	ng/mL	(1.0–1.6)
WBC	3,900	/µL	(3,500–9,800)	GADAb	< 5.0	U/mL	(< 5.0)
RBC	435 × 10^4^	/µL	(376–500)	HBsAg	0.00	IU/mL	(0.00–0.04)
Hemoglobin	13.1	g/dL	(11.3–15.2)	HCVAb	0.1	S/CO	(< 1.0)
Platelet	5.5 × 10^4^	/µL	(13.0–36.9)	AMA2	< 1.5		(< 7)
**Biochemical characteristics**				ANA	20		(< 40)
Total cholesterol	161	mg/dL	(139–220)	Iron	103	µg/dL	(40–188)
Triglycerides	113	mg/dL	(36–149)	TIBC	265	µg/dL	(246–410)
HDL-C	48	mg/dL	(40–87)	Ferritin	121	ng/mL	(5–152)
LDL-C	88	mg/dL	(59–139)	Ceruloplasmin	25	mg/dL	(21–37)
Urea nitrogen	9.7	mg/dL	(8.4–20.4)	M2BPGi	4.27		(< 1.0)
Creatinine	0.50	mg/dL	(0.40–0.74)	4C7S	12.2	ng/mL	(< 4.4)
eGFR	112.2	mL/min/1.73 m^2^		**Endocrinological characteristics**			
Total protein	6.7	g/dL	(6.7–8.2)	LH	< 0.10	mIU/mL	
Albumin	3.6	g/dL	(4.0–4.8)	FSH	< 0.10	mIU/mL	
Total bilirubin	2.0	mg/dL	(0.2–1.2)	Estradiol	24.0	pg/mL	
AST	43	U/L	(10–33)	Growth hormone	< 0.03	ng/mL	(0.13–9.88)
ALT	22	U/L	(6–35)	IGF-1	< 7	ng/mL	(119–283)
ALP	100	U/L	(38–113)	TSH	5.18	µU/mL	(0.61–4.23)
γ-GTP	152	U/L	(8–60)	FT3	1.33	pg/mL	(1.68–3.67)
PT	54.9	%	(70–120)	FT4	0.63	ng/dL	(0.7–1.48)
APTT	38.4	sec	(23.0–39.0)	ACTH	28.8	pg/mL	(7.2–63.3)
Uric acid	3.9	mg/dL	(2.2–6.7)	Cortisol	5.0	µg/dL	(3.7–19.4)
Sodium	142	mEq/L	(135–147)	DHEA-S	33	µg/dL	(58–327)
Potassium	3.6	mEq/L	(3.6–5.0)	Prolactin	12.60	ng/mL	(6.12–30.54)
Chloride	105	mEq/L	(98–108)	**Urinalysis findings**			
Calcium	8.8	mg/dL	(8.8–10.2)	U-CPR	172	µg/day	(29.2–167)
FPG	115	mg/dL	(70–110)	U-Albumin	9	mg/day	(< 30)
Hemoglobin A1c	9.7	%	(4.6–6.2)	U-Copper	13.5	µg/day	(2.5–20.0)
IRI	10.6	µU/mL	(5–10)	U-Cortisol	27.6	µg/day	(5.5–66.7)

Reference ranges are shown in parentheses

*WBC* white blood cell count, *RBC* red blood cell count, *HDL-C* high-density lipoprotein cholesterol, *LDL-C* low-density lipoprotein cholesterol, *eGFR* estimated glomerular filtration rate, *AST* aspartate aminotransferase, *ALT* alanine aminotransferase, *ALP* alkaline phosphatase, *γ-GTP* γ-glutamyl transpeptidase, *PT* prothrombin time, *APTT* activated partial thromboplastin time, *FPG* fasting plasma glucose, *IRI* immunoreactive insulin, *CPR* C-peptide immunoreactivity, *GADAb* anti-glutamic acid decarboxylase antibody, *HBsAg* hepatitis B surface antigen, *HCVAb* hepatitis C virus antibody, *AMA2* anti-mitochondrial M2 antibody, *ANA* antinuclear antibody, *TIBC* total iron binding capacity, *M2BPGi* Mac-2 binding protein glycosylated isomers, *4C7S* type IV collagen 7S, *LH* luteinizing hormone, *FSH* follicle-stimulating hormone, *IGF-1* insulin-like growth factor-1, *TSH* thyroid-stimulating hormone, *FT3* free 3,5,3′-triiodothyronine, *FT4* free thyroxine, *ACTH* adrenocorticotropic hormone, *DHEA-S* dehydroepiandrosterone sulfate

**Table 2:** Results of patient’s pituitary stimulation tests

LHRH stimulation test		0 min	30 min	60 min	90 min	120 min			
LH, mIU/mL		< 0.10	0.15	0.13	0.13	0.13			
FSH, mIU/mL		< 0.10	0.16	0.19	0.25	0.28			
Insulin tolerance test		0 min	15 min	30 min	45 min	60 min	75 min	90 min	120 min
GH, ng/mL	(0.13–9.88)	< 0.03	0.04	0.07	0.10	0.08	0.05	0.05	0.05
ACTH, pg/mL	(7.2–63.3)	25.3	25.2	26.3	112.2	253.1	128.9	79.9	47.1
Cortisol, µg/dL	(3.7–19.4)	4.1	5.3	4.2	7.1	13.7	14.7	13.8	10.6
Glucose, mg/dL	(70–110)	103	85	50	37	127	89	55	66
GHRP-2 stimulation test		0 min	15 min	30 min	45 min	60 min			
GH, ng/mL	(0.13–9.88)	< 0.03	0.05	0.04	< 0.03	< 0.03			
TRH stimulation test		0 min	30 min	60 min	90 min	120 min			
TSH, µU/mL	(0.61–4.23)	3.83	36.23	36.80	35.22	29.06			
PRL, ng/mL	(6.12–30.54)	12.83	31.73	19.84	17.86	15.82			
CRH stimulation test		0 min	30 min	60 min	90 min	120 min			
ACTH, pg/mL	(7.2–63.3)	17.7	95.5	62.8	39.0	33.5			
Cortisol, µg/dL	(3.7–19.4)	6.5	14.8	14.3	12.1	10.8			
Rapid ACTH test		0 min	30 min	60 min					
Cortisol, µg/dL	(3.7–19.4)	4.6	15.3	17.6					

Reference ranges are shown in parentheses

*LHRH* luteinizing hormone releasing hormone, *LH* luteinizing hormone, *FSH* follicle-stimulating hormone, *GH* growth hormone, *ACTH* adrenocorticotropic hormone, *GHRP-2* growth hormone releasing peptide-2, *TRH* thyrotropin-releasing hormone, *TSH* thyroid-stimulating hormone, *PRL* prolactin, *FT4* free thyroxine, *CRH* corticotropin-releasing hormone

Her lack of secondary sexual characteristics made us suspect that genetic abnormalities may have contributed to her pituitary hormone deficiency. We analyzed the coding and splicing regions of 17 major genes associated with congenital hypopituitarism (*HESX1*, *LHX3*, *LHX4*, *OTX2*, *POU1F1*, *PROKR2*, *PROP1*, *SOX2*, *SOX3*, *CHD7*, *FGF8*, *FGFR1*, *GLI2*, *IGSF1*, *KISS1R*, *SOX10*, and *WDR11*) using the NextSeq Sequencing System (Illumina, San Diego, CA, USA) at the Kazusa DNA Research Institute (Kisarazu, Japan) [21]. As a result, a heterozygous missense mutation (c.6745G > A, p.Asp2249Asn) in the *CHD7* gene was identified. This mutation had not been reported in ClinVar and the CHD7 database (www.chd7.org), and the minor allele frequency was < 0.1% in several population databases (0.0011% in Genome Aggregation Database v2.1.1, 0.0015% in Trans-Omics for Precision Medicine, 0% in the Human Genetic Variation Database, and 0.099% in the Japanese Multi Omics Reference Panel [14KJPN]). The nucleotide position showed a phyloP conservation score [22] of 7.526 for 100 vertebrates, a PhastCons score [22] of 1.00 for 100 vertebrates, and a Genomic Evolutionary Rate Profiling score [23] of 5.3 for mammalian alignment, indicating that this mutation occurred in a highly conserved nucleotide. Numerous in silico prediction tools such as Polymorphism Phenotyping v2 [24], MutationTaster [25], Functional Analysis through Hidden Markov Models [26], Mendelian Clinically Applicable Pathogenicity [27], and Combined Annotation-Dependent Depletion [28] (CADD score, 25.4) suggested that this mutation causes changes in protein function, although the mutation was considered to have uncertain significance according to the American College of Medical Genetics and Genomics (ACMG) guidelines [29]. Taken together, the *CHD7* missense mutation in this patient was suggested to be pathogenic and contributory to the CPHD.

The patient underwent ophthalmologic and otorhinolaryngologic examinations, brain MRI, echocardiography, abdominal MRI, and EGD for the evaluation of CHARGE features. Apart from CPHD and intellectual disability, there was no evidence of CHARGE or CHARGE-like features such as coloboma of the eyes, atresia of choanae, anomalies of the semicircular canals, external or middle ear anomalies, hearing loss, hyposmia, olfactory bulb hypoplasia, cranial nerve dysfunctions, cleft lip/palate, anomalies of the mediastinal viscera, and renal anomalies.

## Discussion and conclusions

Herein, we present the clinical history of a 33-year-old woman with CPHD (HH, GHD, and CH) alongside comorbid diabetes, severe obesity, and liver cirrhosis probably due to NASH. Despite not fulfilling the criteria for CHARGE syndrome, the patient had intellectual disability, one of its minor features, and a *CHD7* missense mutation with pathogenic potential.

Whether the *CHD7* mutations can be detected in patients with CPHD who do not satisfy the criteria for CHARGE syndrome remains to be elucidated. Recently, a synonymous *CHD7* mutation predicted to affect splicing had been identified in one of 80 patients with CPHD who underwent a comprehensive genetic examination [30]. However, this report does not include a detailed description of CHARGE features. Several studies have highlighted the importance of careful evaluation of CHARGE features in patients with *CHD7* mutations, as a subset of patients with apparently isolated HH harboring *CHD7* mutations were subsequently found to have multiple CHARGE features and were reclassified as having CHARGE syndrome [31–33]. Our patient is a rare case of CPHD harboring a *CHD7* mutation that, after detailed examinations, does not certainly fulfill the criteria for CHARGE syndrome.

**Fig. 3:**
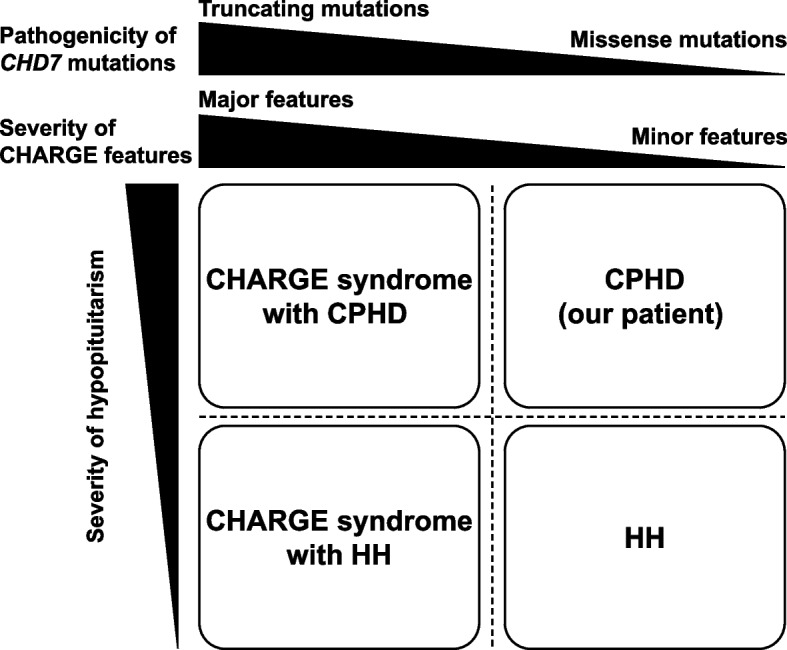
The concept of CHD7-associated syndrome. *CHD7* mutations can have a continuous phenotypic spectrum depending on the severity of hypopituitarism and CHARGE features. HH, congenital hypogonadotropic hypogonadism; CHD7, chromodomain-helicase-DNA-binding protein 7; CPHD, combined pituitary hormone deficiency

In patients with isolated HH harboring *CHD7* mutations without CHARGE syndrome, some patients have minor CHARGE features, such as hearing loss or intellectual disability [11, 15–20]. In addition, *CHD7* mutations in CHARGE syndrome are typically truncating and can lead to serious genetic dysfunction, whereas mutations in isolated HH with or without minor CHARGE features are predominantly of the missense type that has less impact on gene function compared with truncating mutation [15, 16]. Furthermore, following the classification of missense mutations in patients with isolated HH based on ACMG guidelines, minor CHARGE features have been reported to be more commonly present in patients with pathogenic or likely pathogenic mutations than in those with uncertain significance mutations [20]. These pieces of evidence suggest that isolated HH harboring *CHD7* mutations is a mild form of CHARGE syndrome. Given that our patient had intellectual disability, which is a minor feature of CHARGE syndrome, and that the *CHD7* mutation was of the missense type, our patient with CPHD may also be considered as having a mild form of CHARGE syndrome. Moreover, based on the above findings, including those of our patient, we speculate that *CHD7* mutations may have a continuous phenotypic spectrum depending on the severity of hypopituitarism and CHARGE features, and propose a novel concept of CHD7-associated syndrome, including CHARGE syndrome (Fig. 3).

Previous studies have indicated that HH caused by *CHD7* mutations is due to hypothalamic gonadotropin-releasing hormone (GnRH) deficiency resulting from the impaired migration of GnRH-synthesizing neurons that migrate alongside the olfactory fibers during embryonic development [11, 34]. This postulated etiology is consistent with the observations that most individuals with CHARGE syndrome have olfactory dysfunction and do not have abnormalities in other anterior pituitary hormone levels or pituitary morphology. However, a subset of individuals with CHARGE syndrome can have CPHD [5–9, 12, 13], and some of these patients can demonstrate structural pituitary abnormalities such as anterior pituitary hypoplasia [13], similar to that observed in our patient. CHD7 is expressed in the olfactory epithelium associated with the migration of GnRH neurons as well as the developing anterior pituitary and hypothalamus in both mice and humans [35, 36]. CHD7 has also been identified as a SOX2 transcriptional cofactor [37]. In addition, recent studies have shown that CHD7-deficient mice have hypoplastic Rathke’s pouches [38] and reduced OTX2 mRNA expression [39]. Both SOX2 and OTX2 are associated with pituitary development and differentiation [40, 41]. These pieces of evidence suggest that CHD7 potentially plays a role in the development and function of the hypothalamic-pituitary axis and that *CHD7* mutations can result in variable degrees of hypopituitarism, as well as HH. Further studies are needed to elucidate more detailed mechanisms underlying the relationship between CHD7 and the hypothalamic-pituitary axis.

Our patient had experienced short stature until approximately 15 years of age, after which she had grown taller and had attained normal height despite GHD. Growth without GH has been reported in rare cases of hypopituitarism untreated until adulthood [42–44]. Hyperinsulinemia, hyperleptinemia, or delayed epiphyseal maturation caused by concomitant HH have been postulated as possible underlying mechanisms [44, 45]. Several factors such as endocrine, paracrine, intracellular, or extracellular matrix factors may also contribute to normal growth in GHD [46]. In fact, previous evidence has shown that the GH–insulin-like growth factor-1 (IGF-1) axis is just one of many regulatory systems that control height [46].

In our patient, long-term untreated GHD and CH may have contributed to the development of diabetes, severe obesity, and liver cirrhosis probably due to NASH [47]. In particular, GHD can induce NASH, and liver fibrosis is associated with a lower IGF-1 level even in adolescents and young adults with GHD [48]. GH replacement therapy improves liver dysfunction and histological hepatic characteristics, including steatosis and fibrosis, as well as metabolic abnormalities [49, 50]. However, it is unclear to what extent GH replacement therapy would improve the liver function of our patient because of the severe advanced cirrhosis. Therefore, we will carefully monitor the effect of the treatment on her liver function.

The present case report had several limitations. First, we could not perform genetic analyses on the proband’s parents. Most *CHD7* truncating mutations in typical CHARGE syndrome are heterozygous de novo, whereas *CHD7* missense mutations in isolated HH are often inherited from unaffected parents, suggesting incomplete penetrance of the phenotype of pituitary hormone deficiencies in individuals harboring *CHD7* missense mutations [15–18]. Thus, the *CHD7* mutation observed in our patient may be inherited from either unaffected parent. Second, a limited number of genes associated with pituitary hormone deficiencies were analyzed. Although various genes associated with congenital pituitary hormone deficiencies have been identified, a majority of the related genes remain unknown [51]. In addition, oligogenic inheritance has been observed in a number of cases of isolated HH [16–18, 52]. Therefore, some unknown gene mutations in addition to the observed *CHD7* mutation may contribute to the phenotype of our patient in an additive or synergistic manner, and the oligogenicity may explain the incomplete penetrance or phenotypic variations in patients harboring *CHD7* mutations. Third, the observed *CHD7* mutation was not confirmed by Sanger sequencing. However, the next-generation sequencing workflow we employed has been shown to have high specificity and can omit the need for confirmatory assessment by Sanger sequencing for variants with high-quality scores [21]. Finally, as is the case with most previously reported cases of CHD7 missense mutations, we were unable to confirm the functional abnormality of the observed *CHD7* mutation via in vitro or in vivo experiments. Despite these limitations, the findings that our patient’s *CHD7* mutation was an ultra-rare variant (< 0.1%), occurred at a highly conserved nucleotide, and was predicted to be damaging by numerous major bioinformatic tools, strongly suggest that this missense mutation was associated with her phenotype.

In conclusion, we report a rare case of CPHD with a minor feature harboring a *CHD7* mutation. This case does not only imply that CHD7 plays a potential role in the development and function of the hypothalamic-pituitary axis but provides valuable insights into phenotypes caused by *CHD7* mutations. *CHD7* mutations can have a continuous phenotypic spectrum depending on the severity of hypopituitarism and CHARGE features. Therefore, we would like to propose a novel concept of CHD7-associated syndrome.

## Data Availability

The datasets generated and/or analysed during the current study are available in the DDBJ repository, accession number DRA014912.

## Abbreviations

CHD7: Chromodomain helicase DNA-binding protein 7
HH: Hypogonadotropic hypogonadism
CPHD: Combined pituitary hormone deficiency
GH: Growth hormone
TSH: Thyroid-stimulating hormone
ACTH: Adrenocorticotropic hormone
NASH: Nonalcoholic steatohepatitis
GHD: Growth hormone deficiency
CH: Central hypothyroidism
TRH: Thyrotropin-releasing hormone
ITT: Insulin tolerance test
CRH: Corticotropin-releasing hormone
MRI: Magnetic resonance imaging
EGD: Esophagogastroduodenoscopy
ACMG: American college of medical genetics and genomics
GnRH: Gonadotropin-releasing hormone
IGF-1: Insulin-like growth factor-1
